# Spatiotemporal evolution patterns of the coupling of carbon productivity and high-quality economic development in China

**DOI:** 10.1038/s41598-025-92044-2

**Published:** 2025-03-17

**Authors:** Lingdi Li, Mohammad Affendy Arip, Puah Chin Hong

**Affiliations:** 1 Faculty of Economics and Business, Universiti Malaysia Sarawak, Kota Samarahan, 94300 Malaysia; 2 Anhui Sanlian University, 230061 Hefei, China

**Keywords:** Carbon productivity, High-quality economic development, Coupling coordination degree, Spatiotemporal analysis, Spatial effect analysis, Environmental economics, Socioeconomic scenarios, Sustainability

## Abstract

This study examines the spatiotemporal evolution of the coordinated development between carbon productivity (CP) and high-quality economic development (HQED) across 30 provinces in China from 2008 to 2021. Using the entropy weight method, coupling coordination degree (CCD), kernel density estimation, spatial autocorrelation analysis, and spatial econometric models, the research identifies several key findings: first, a coupling and coordination relationship characterized by mutual influence and restraint exists between carbon productivity and high-quality economic development. Both carbon productivity and high-quality economic development, along with their coupling coordination degree, have exhibited continuous growth, demonstrating a spatial distribution pattern of “higher in the east than in the west, and higher in the south than in the north,” accompanied by expanding spatial concentration and pronounced regional disparities. Second, the global Moran’s I for the coupling coordination degree is positive, indicating significant spatial effects between carbon productivity and high-quality economic development. The LISA map highlights that high–high clusters are concentrated in the economically advanced eastern coastal areas, while low–low clusters are predominantly located in underdeveloped central and western regions and energy-dependent heavy industrial provinces. Third, the spatial effects of coupling coordination degree are influenced by factors such as economic development level, urbanization, technological progress, environmental regulation, the proportion of the secondary industry, and marketization level. The significance of these factors varies in the decomposition effect. Finally, this study provides policy recommendations. Within the framework of China’s “dual-carbon” goals, promoting the coupling and coordinated development of carbon productivity and high-quality economic development, while fostering balanced regional growth, holds substantial practical importance.

## Introduction

The ecological environment and socio-economic systems are intricately linked, constituting a complex coupled system where mutual constraints and integration occur^1^. Harmonizing their relationship is crucial for survival and sustainable development of human society. The 20th National Congress Report emphasizes that “promoting the greening and low-carbon transformation of economic and social development is essential for achieving high-quality development”^2^. Green and low-carbon development necessitates a win–win situation between economic growth and environmental protection, with CP serving as a crucial link between the two. As climate change emerges as a significant global challenge, exploring the coordinated development of CP and HQED has become central to China’s sustainable development strategy. HQED is a development model that takes innovation as the primary driving force, coordination as an inherent feature, green development as a universal form, openness as a necessary path, and shared prosperity as the fundamental goal. It is not only an adjustment of the economic growth rate but also a profound transformation of the development approach, economic structure, and driving forces. HQED provides the policy support and market environment needed to enhance CP, while improving CP aids HQED by reducing carbon emissions and boosting economic efficiency through technological innovation and industrial upgrading.

Kaya and Yokobori^3^ first proposed the concept of CP to represent the economic benefits derived from each unit of carbon emissions. It serves as a key indicator for measuring economic output per unit of carbon emissions and is essential for evaluating both environmental and economic performance^4^. Evaluating carbon productivity enables the assessment of a country’s contribution to addressing global climate change^5^.

**Fig. 1:**
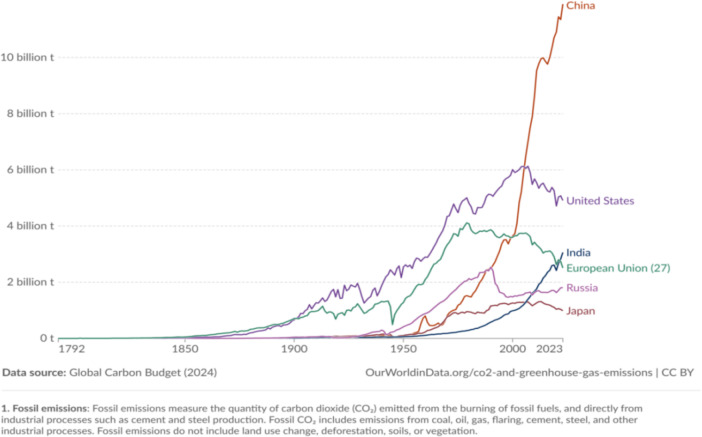
Top CO_2_ emitting countries in the world (1792–2023).

Currently, climate change is intensifying, primarily driven by excessive fossil fuel consumption, which is the leading cause of greenhouse gas emissions. These emissions drive global warming and pose significant environmental risks^6^. Notably, CO_2_ emissions accounting for over 80% of total greenhouse gas emissions^7^, are central to climate change mitigation efforts. Among the world’s major carbon emitters, China is the largest source of CO_2_ emissions (Fig. 1), primarily due to its status as the largest industrialized developing country. This ongoing rise in emissions highlights the immense pressure China faces in balancing economic growth with environmental challenges. In September 2020, President Xi Jinping announced at the 75th session of the United Nations General Assembly (UNGA) that China aims to peak CO_2_ emissions before 2030 and achieve carbon neutrality before 2060. Simultaneously, China seeks to build a modern socialist country that is prosperous, strong, democratic, culturally advanced, harmonious, and beautiful. These two national strategic goals present both unprecedented challenges and significant opportunities. Enkvist et al.^8^ proposed that improving CP can decouple economic growth from carbon emissions, facilitating the transition to a low-carbon economy. China can mitigate global climate change by improving CP^9^.

Therefore, scientifically assessing the CCD between CP and HQED as well as its spatiotemporal evolution pattern, provides valuable insights for enhancing the coordinated development of CP and HQED. This has significant practical implications for achieving the ‘dual carbon’ goals and fostering high-quality development.

## Literature review

### Relationship between carbon productivity and economic development

Wang et al.^10^ concluded that economic development positively influences CP, with this effect strengthening as economic growth increases. Furthermore, the relationship between economic growth and CP is highly nonlinear. Qi et al.^11^, using panel data from BRICS and G7 countries, analyzed economic growth patterns by integrating CP and economic growth, confirming the feasibility of low-carbon economic growth models. Moreover, promoting economic agglomeration, coordinating urban cluster development, and implementing fiscal policies can enhance carbon productivity and generate spillover effects in neighboring cities^12,13^. The study of this relationship can be examined from temporal and spatial dimensions.

At the temporal level, research methods primarily include the Environmental Kuznets Curve (EKC), decoupling theory, integrated approaches combining decoupling theory with other methods, and causal analysis. The EKC illustrates a potential pathway for sustainable economic development^14^. Building on the EKC framework, international scholars have introduced the Carbon Emissions Kuznets Curve (CEKC). While most researchers agree on the existence of the CEKC, its specific shape remains debated, with hypotheses including “U-shaped,” “inverted U-shaped,” “N-shaped,” and “inverted N-shaped” curves^15–17^.

Decoupling theory describes the dynamic relationship between economic development and environmental pollution, emphasizing whether the two variables change synchronously. Gbadeyan et al.^18^ emphasize that achieving a low-carbon economy and supporting carbon reduction strategies require decoupling economic growth from carbon emissions growth. Economic growth remains the primary driver of global carbon emissions, and decoupling is essential for achieving environmental sustainability.

Iorember et al.^19^ utilized the Cross-Sectional Augmented Autoregressive Distributed Lag (C-S ARDL) method and the Tapio decoupling index to evaluate the decoupling status of BRICS countries, uncovering varying decoupling states among them. Numerous scholars have applied the Tapio decoupling index and the LMDI decomposition model, concluding that decoupling is achievable^20–22^.

At the spatial level, research methods primarily include spatial association and agglomeration analysis, Kernel density estimation, geographic weighted regression, CCD model, spatial econometric models, and their integration with Geographic Information Systems (GIS) to merge data visualization with analytic methods. Spatial econometric models are widely employed to analyze spatial dependence and heterogeneity in spatial data, focusing the spillover effects of regional economic activities or carbon emissions on neighboring areas^23–25^. Spatial association and clustering analysis investigate spatial autocorrelation, revealing relationships between neighboring regions and identify spatial clustering patterns^26^. owing to its simplicity, intuitive results, and broad applicability, the CCD model has become an effective tool for examining interactions and coordinated development between systems^27,28^ However, researchers should exercise caution to avoid common errors and misapplications^29^. Tools common used to integrate spatial econometric models with GIS include GeoDa, ArcGIS, and MATLAB, facilitating the fusion of data visualization and analytical methods^30^.

The spatial weight matrix plays a crucial role in spatial analysis. Pijnenburg and Kholodilin^31^ highlighted the critical role of an appropriate weight matrix, emphasizing that the primary advantage of spatial analysis lies in spillover effects, which are largely determined by the choice of the weight matrix. Indirect effects are particularly sensitive to the specifications of neighboring areas. Thus, the spatial weight matrix is a fundamental component of spatial analysis and requires careful definition. Improperly specifying adjacency relationships can lead to significantly distorted and inaccurate results^32^.

The aforementioned methods have collectively established a robust theoretical foundation for this research.

### Coupling coordination between carbon productivity and high-quality economic development

China has transitioned from relying solely on GDP to employing a multidimensional approach to evaluate the quality of its economic development. Resolving the conflict between economic growth and carbon emissions is essential to achieving high-quality economic development. Limited studies have focused on the coupling coordination analysis of CO_2_ emissions and HQED. This analysis typically explores CO_2_ emission intensity or efficiency in relation to HQED. For instance, studies have examined the spatiotemporal changes and factors influencing the coupling coordination between agricultural, marine, and land carbon emission efficiency and their relationship with HQED^33,34^. Another approach investigates the relationship between regional CO_2_ emission intensity and HQED. For example, CO_2_ emission intensities in the Yangtze River Delta, the middle and lower reaches of the Yangtze River, and the Yellow River Basin are interconnected and aligned with HQES, as seen in provinces like Guangdong and Hubei^35,36^.

### Literature evaluation

Current research has primarily focused on the relationship between carbon emissions and HQED. emphasizing specific indicators or dimensions, while analyzing unidirectional impacts and coupling relationships. Studies analyzing the coupling and coordination relationship between carbon emission and HQED a at the national provincial level remain relatively scarce. China’s economy is a complex system characterized by the interdependence, interaction, and mutual constraints of social, economic, and ecological components, resulting in imbalanced development trend. examining the coupling relationship with carbon emissions from a single region or industry, or through limited dimensions, fails to accurately reflect the broader context. Furthermore, this approach overlooks regional characteristics of China’s economic development and the effectiveness of policy implementations.

Existing studies have primarily relied on indicators such as carbon intensity or efficiency to evaluate harmonization with HQED. However, these indicators fail to comprehensively capture the coordination between China’s economic development and regional agglomeration. When comprehensive indicators are used, carbon emission efficiency may fail to accurately reflect CO_2_ production efficiency, potentially contradicting the “dual-carbon” goal. Moreover, carbon emission intensity shows an inverse relationship with HQED, with unsatisfactory coupling and coordination effects.

In the existing literature, some studies fail to specify which spatial weight is used. Some simply employ the adjacency spatial weight matrix, while others utilize the weight matrix provided by the econometric software. All of these situations can lead to unreasonable analyses of spatial correlation.

The potential marginal contributions of this paper lie in three aspects:

This study employs the CP indicator as it aligns with the trajectory of HQED. CP embodies mutual influence, promotion, and coordination. This research identifies trends and effects across temporal and spatial dimension as CP and HQED progress in a coordinated manner.

The economic-geographic spatial weight matrix is selected. This matrix integrates economic and geographical factors, dynamically adjusting spatial weights according to regional economic-geographic characteristics^37,38^, thereby effectively capturing the spatial distribution patterns of CP and HQED.

**Fig. 2:**
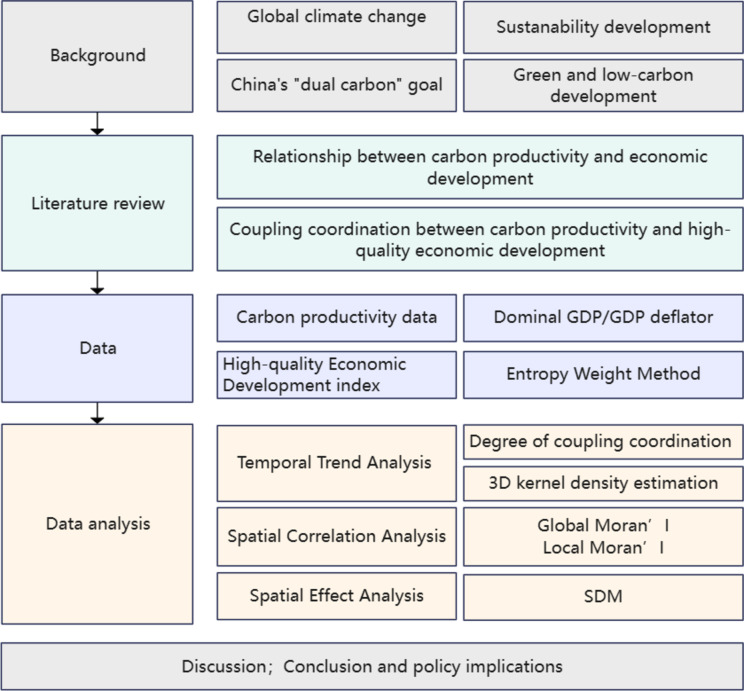
Content framework. *Source*: Authors’ own illustrations.

This study addresses a gap in the literature by providing an in-depth analysis of the temporal evolution of the relationship between carbon productivity and high-quality economic development. This paper offers a valuable reference for policy formulation and coordination across regions, promoting sustainable economic development and advancing ecological civilization. The content framework of this study is illustrated in Fig. 2.

## Methods and data

### Measuring carbon productivity

The existing literature on CO_2_ assessment addresses multiple perspectives, including total carbon emissions, intensity, performance indicators, and implied carbon emissions. Numerous studies focus on provincial, municipal, or industry data. Given data reliability, this study employs data from the Carbon Emission Accounts and Datasets (CEADs)^39–41^.

Calculating CP requires annual GDP data for each province, as well as the provincial and municipal GDP statistics from the *China Statistical Yearbook*. This study employs the GDP index method to calculate each province’s real GDP, using 2008 as the base year to ensure result comparability across years. The calculated formula is defined as follows:

1 $$CP_{it} = GDP_{it} /CE_{it}$$

where* i* and *t* represent the city and year, respectively.

### Measuring high-quality economic development

Two principal methods exist for measuring the level of HQED. One approach volves using single indicator, such as labor productivity, total factor productivity, green total factor productivity, the contribution rate of technological progress to economic growth, or welfare carbon emission intensity^42–44^. Another method involves creating composite indicators. These indicators are created using methods such as the relative index, hierarchical analysis, entropy value, and factor analysis. HQED is a multidimensional evaluation index^45–47^. This paper adopts Zhao’s (2020) perspective, defining HQED as encompassing three dimensions: economic foundation, social foundation, and ecological foundation. It establishes a multidimensional evaluation system for measuring HQED, consisting of six secondary indicators: industrial structure, technological innovation, inclusive total factor productivity (TFP), openness to the outside world, residents’ livelihoods, and ecological environment. The composition of the specific indicators is detailed in Table 1.

**Table 1:** Indicators of high-quality economic development.

Fundamentals	Primary indicators	Second indicators (weight)	Indicator description	Abbreviation (attributes)
Economic fundamentals	Industry structure (0.1407)	Percentage of productive service industry (0.0389)	Proportion of productive service industries in urban employment	PPS (+)
		Rationalization of industrial structure (0.0350)	Structural deviation method	RIS (+)
		Heightened industrial structure (0.0668)	Ratio of the output value of the tertiary sector to the secondary sector	HIS (+)
	Technological innovation (0.2099)	innovation index (0.2099)	Innovation index at the urban level	IN (+)
	Inclusive total factor productivity (0.0346)	Inclusive TFP (0.0346)	Calculation using the Hicks-Moorsteen index method	TFP (+)
	Openness to the outside world (0.4581)	Foreign trade openness (0.3580)	Foreign direct investment / regional GDP	FDO (+)
		Openness to foreign investment (0.1001)	Total imports and exports / regional GDP	OFI (+)
Social fundamentals	Residents’ Living Standards (0.1228)	Per Disposable personal income (0.0481)	Regional GDP / total resident population	PDPI (+)
		Expenditure on education per capita (0.0452)	Local government financial education expenditure / total resident population	PCE (+)
		Hospital beds per capita (0.0295)	Number of hospital beds / total resident population	PHB (+)
Ecological fundamentals	Ecodevelopment (0.0543)	SO_2_ emissions per unit (0.0008)	SO_2_ / regional GDP	SEP (−)
		Comprehensive utilization rate of industrial solid waste (0.0389)	Comprehensive utilization / total output + storage volume	CUI (+)
		PM2.5(0.0146)	Population-weighted PM2.5 concentration	PM (−)

#### Further elaboration on the indicators

Industry structure. The transformation and upgrading of the industrial structure are key driving force for HQED. The selection of indicators mainly focuses on the advancement and rationalization of the industrial structure. Moreover, producer services, as a core component of the ongoing technological revolution and industrial transformation, are included. The rationalization of the industrial structure is calculated using the structural deviation method^48^. The calculation formula is defined as:

2 $$E = \sum\limits_{i = 1}^{{\text{n}}} {\left| {\frac{{{\raise0.7ex\hbox{${Y_{i} }$} \!\mathord{\left/ {\vphantom {{Y_{i} } Y}}\right.\kern-0pt} \!\lower0.7ex\hbox{$Y$}}}}{{{\raise0.7ex\hbox{${L_{i} }$} \!\mathord{\left/ {\vphantom {{L_{i} } L}}\right.\kern-0pt} \!\lower0.7ex\hbox{$L$}}}} - 1} \right|}$$

*E:* industrial structure deviation. *Y:* total output value. L: total employment. *n:* number of industrial sectors. *i:* Individual industrial sectors (primary, secondary, and tertiary).

Technological Innovation is a critical driver of HQED and a decisive factor in urban economic development quality. It exerts a direct and significant impact on economic development quality. This study uses the urban-level innovation index from the *China Urban and Industry Innovation Report 2017* to measure innovation levels^49^.

Inclusive TFP. Efficiency and equity in economic growth are fundamental dimensions for evaluating economic development quality. Capital and labor are incorporated as inputs, real GDP as expected output, and the urban-rural income gap as non-expected output to calculate TFP using the Hicks-Moorsteen index method. The Hicks-Moorsteen index is constructed using the Shephard distance function. The input and output distance functions are expressed as follows:

3 $$\begin{aligned} & Y(y) = \left[ {D_{0} (x_{hs} ,y,s)D_{0} (x_{it} ,y,t)} \right]^{1/2} \\ & X(x) = \left[ {D_{1} (x,y_{hs} ,s)D_{1} (x,y_{it} ,t)} \right]^{1/2} \\ \end{aligned}$$

X and y represent the vectors of input and output data, respectively; s and t denote time; D_1_(x, y) is the input Shephard distance function, and D_0_(x, y) is the output Shephard distance function^50^ then:

4 $$TFP_{hs,it} = \left\{ {\frac{{D_{0} (x_{hs} ,y_{it} ,s)D_{1} (x_{hs} ,y_{hs} ,s)D_{0} (x_{it} ,y_{it} ,t)D_{1} (x_{hs} ,y_{it} ,t)}}{{D_{0} (x_{hs} ,y_{hs} ,s)D_{1} (x_{it} ,y_{hs} ,s)D_{0} (x_{it} ,y_{hs} ,t)D_{1} (x_{it} ,y_{it} ,t)}}} \right\}^{{ ^{1/2} }}$$

The data processing and methods involved in this formula refer to these two papers^51^

*Openness to the outside world*. China’s reform and opening-up over the past 40 years has demonstrated that expanding openness to the outside world is a vital driver of economic and social development and a fundamental pathway to national prosperity and strength. Indicators such as trade dependence and foreign direct investment (FDI) openness effectively reflect the degree of economic openness and international competitiveness, offering a holistic assessment of high-quality economic development.

*Residents’ living standards*. The primary objective of high-quality economic development is to improve the well-being of the population. Thus, per capita disposable income, per capita education expenditure, and per capita hospital bed availability are included as indicators representing the dimension of residents’ living standards.

*Ecodevelopment*. The construction of ecological civilization is both a crucial foundation for high-quality development and a significant indicator of its achievements. Since the 18th National Congress of the Communist Party of China, China’s economic development strategy has prioritized green development principles. The three selected indicators effectively reflect urban environmental quality, resource utilization outcomes, and pollution control achievements.

### Selection of influencing factors

The coupling and coordinated development of CP and HQED is shaped by various factors. These factors encompass social, economic, ecological, and spatial dimensions, including regional heterogeneity and spillover effects. Research on the coupling coordination mechanisms between the two remains in the exploratory stage, with limited focus on the spatial effects of their interaction. Some studies have examined the driving factors influencing the coupling and coordination of carbon emission intensity, carbon emission efficiency, and high-quality economic development, using methods such as factor analysis and geographic detectors^52,53^.

Building on existing research, this study selects six variables—economic development, industrial structure, urbanization, marketization level, technological innovation, and environmental regulation—to construct a spatial econometric model for analyzing the influencing factors and spatial effects of the CCD. The selected variables do not overlap with those used to construct the HQED indicators. furthermore, considering the uncertainties of the current international political and economic landscape, the variables primarily focus on internal driving factors.

(1) GDP per capita (*pgdp*) indicates the level of economic development; (2) Environmental regulation (*er*) is assessed by the investment in industrial pollution control per thousand yuan of industrial added value; (3) Research and Development (*rd*) investment promotes green productivity and is measured by the proportion of R&D expenditure of large-scale industrial enterprises in regional GDP; (4) The urbanization rate (*ur*) is calculated as the ratio of urban population to the total resident population; (5) the marketization level (*ml*) reflects the ownership structure of China’s economy and is calculated as the proportion of employment in private enterprises and individual employment to total employment; (6) Industrial structure (*si*) enhances resource allocation and production efficiency and is measured as the proportion of secondary industry output value in GDP.

### Data sources

Most of the data used in the study are sourced from the website of *the National Bureau of Statistics of the People’s Republic of China, the Energy Statistics Yearbook, the Statistical Bulletin of National Economic and Social Development, the Industrial Statistics Yearbook of China, the Environmental Statistics Yearbook of China, the Statistical Yearbook of China* for the period 2008–2021.

## Methods

### Entropy weight method

#### Data standardization

Suppose there are *m* evaluated objects, each with *n* indicators. consequently, an $m\times n$ judgment matrix can be constructed. after standardize indicators, a new matrix $R=({{r}_{ij})}_{m\times n}.$ ${r}_{ij}\in \left[\text{0,1}\right]$ is obtained, the formula is given as follows:

5 $$X = (r_{ij} )_{m \times n} (i = 1,{ }2, \ldots ,{ }m;j{ } = { }1,{ }2,{ } \ldots { },{ }n)$$

If the indicator has a positive trend,

6 $$r_{ij} = \frac{{x_{ij} - \min x_{ij} }}{{\max x_{ij} - \min x_{ij} }}$$

If the indicator has a negative trend,

7 $$r_{ij} = \frac{{\max x_{ij - } x_{ij} }}{{\max x_{ij} - \min x_{ij} }}$$

Calculation of entropy:

We can calculate the contribution of year *i* under index *j* via the following equation:

8 $$\begin{aligned} & e_{ij} = - k\mathop \sum \limits_{i = 1}^{n} P_{ij} \ln \left( {P_{ij} } \right) \\ & P_{ij} = \frac{{r_{ij} }}{{\mathop \sum \nolimits_{i = 1}^{n} r_{ij} }} \\ & k = \frac{1}{lnn}{ } \\ \end{aligned}$$

Calculation of weight values for indicators

9 $$W_{j} = \frac{{1 - e_{j} }}{{\mathop \sum \nolimits_{j = 1}^{m} 1 - e_{j} }}$$

Calculation of the composite evaluation index for each year:

10 $$S_{i} = \mathop \sum \limits_{j = 1}^{n} W_{j} P_{ij}$$

### Coupling coordination degree model

The CCD of CP and HQED is constructed to analyze their synergistic effect. The specific formulas are as follows:

11 $$C = \frac{{2\sqrt {U_{1} \times U_{2} } }}{{U_{1} + U_{2} }}$$

*C* denote the coupling degree, and ${U}_{1}$ and ${U}_{2}$ represent the composite indices of CP and HQED for each province, respectively:

12 $$\begin{aligned} & T = \alpha U_{1} + \beta U_{2} \\ & D = \sqrt {CT} \\ \end{aligned}$$

where* D* represents the CCD, ranging from 0 to 1; *T* denote the overall scores of the two systems; and α and β represent the contribution shares of CP and HQED, respectively. given that both CP and HQED play equally important roles in social development, *α* = *β* = 0.5. A higher CCD value indicates a strong correlation between CP and HQED, signifying that these two factors are advancing in close coordination. The specific classification is provided in Table 2.

**Table 2:** Coupling coordination degree classification.

Coupled coordination development stage	Type	Range of D value
Incoordination	Severely incoordination	0 ≤ D ≤ 0.1
	Extremely incoordination	0.1 < D ≤ 0.2
	Moderately incoordination	0.2 < D ≤ 0.3
	Mildly incoordination	0.3 < D ≤ 0.4
Adjustment	Critically coordination	0.4 < D ≤ 0.5
	Critically coordination	0.5 < D ≤ 0.6
Coordination	Mildly coordination	0.6 < D ≤ 0.7
	Moderately coordination	0.7 < D ≤ 0.8
	Highly coordination	0.8 < D ≤ 0.9
	Extremely coordination	0.9 < D ≤ 1

### Kernel density estimation

Kernel Density Estimation (KDE), a non-parametric statistical method, effectively captures the distribution characteristics of multidimensional data, offering a more comprehensive analysis. This study applies KDE to analyze the distribution trend of the CCD between CP and HQED. The kernel density function for the random variable *x* is given by:

13 $${\text{f}}(x) = \frac{1}{nh}\sum\limits_{i = 1}^{n} {K\left( {\frac{{x - X_{i} }}{h}} \right)}$$

K(•) is the kernel function, *x*_1_ ~ *x*_n_ represents the coupling coordination level values, *x* is the mean value, *n* denotes the number of observations, and *h* is the window width^54^. This study employs the Gaussian kernel function, known for its higher accuracy, to estimate the dynamic distribution characteristics of CP, HQED, and their CCD.

### Exploratory spatial data analysis

Exploratory spatial data analysis (ESDA) is a method used to explore the distribution and relationships of spatial data^55^. ESDA operates under the assumption that nearby locations tend to exhibit similar attributes, with these similarities diminishing as distance increase. It considers both global and local autocorrelation analysis. The formula for global autocorrelation is as follows:

14 $$I = \frac{{\sum\limits_{i = 1}^{n} {\sum\limits_{j = 1}^{n} {W_{ij} \left( {x_{i} - \overline{x}} \right)\left( {x_{j} - \overline{x}} \right)} } }}{{\sum\limits_{i = 1}^{n} {\left( {x_{i} - \overline{x}} \right)^{2} } }}$$

where ${x}_{i}$ and ${x}_{j}$ represent the observations of region* i* and region *j*, respectively; *x* is the mean value of all sample observations within the study area; *n* denotes the number of cities; and ${W}_{ij}$ represents the spatial adjacency matrix. which indicates the spatial proximity of cities. Moran’s I ranges from − 1 to 1, where values below 0 indicate negative spatial correlation, a value of 0 indicates no spatial correlation, and values above 0 indicate positive spatial correlation^56^.

Global Moran’s I examines the overall spatial autocorrelation within an entire region but fails to capture spatial heterogeneity and specific clustering patterns between local areas. Local Moran’s I effectively addresses this limitation and can be measured via the following formula^36^:

15 $$I_{i} = \frac{{n(x_{i} - \overline{x})\sum\limits_{j = 1}^{n} {W_{ij} (x_{j} - \overline{x})} }}{{\sum\limits_{i = 1}^{n} {\left( {x_{i} - \overline{x}} \right)^{2} } }}$$

### Spatial econometric model

In studying the coupling and coordination relationship between CP and HQED, the spatial association must be considered when analyzing its influencing factors. Therefore, a spatial econometric model is employed as an effective tool to analyze the influencing factors and spatial effects. The Spatial Durbin Model (SDM) combines the advantages of the Spatial Autoregressive Model (SAR) and the Spatial Error Model (SEM), fully accounting for spatial dependence and spatial heterogeneity, thus offering a more comprehensive spatial perspective. Based on this, the SDM for the influencing factors of the coupling and coordination relationship between CP and HQED is formulated as follows:

16 $$\begin{aligned} & D_{it} = \beta_{0} + \rho \omega D_{it} + \beta_{1} pgdp_{it} + \beta_{2} er_{it} + \beta_{3} ur_{it} + \beta_{4} rd_{it} + \beta_{5} ml_{it} + \beta_{6} si_{it} \\ & \quad + \lambda_{1} \omega pgdp_{it} + \lambda_{2} \omega er_{it} + \lambda_{3} \omega ur_{it} + \lambda_{4} \omega rd_{it} + \lambda_{5} \omega ml_{it} + \lambda_{6} \omega si_{it} + \mu_{i} + \nu_{t} + \varepsilon_{it} \\ \end{aligned}$$

In the formula, *D*_*it*_ represents CCD of CP and HQED, ρ is the spatial spillover coefficient, ω is the spatial weight matrix, β_1_ ~ β_7_ are the regression coefficients for the explanatory variables. λ_1_ ~ λ_7_ denote the spatial spillover coefficients for each explanatory variable, *μ*_*i*_ and* ν*_*t*_ are the spatial effect and time effect, respectively, and* ε*_*it*_ is the random disturbance term.

## Results

### Spatiotemporal evolution patterns of carbon productivity

**Fig. 3:**
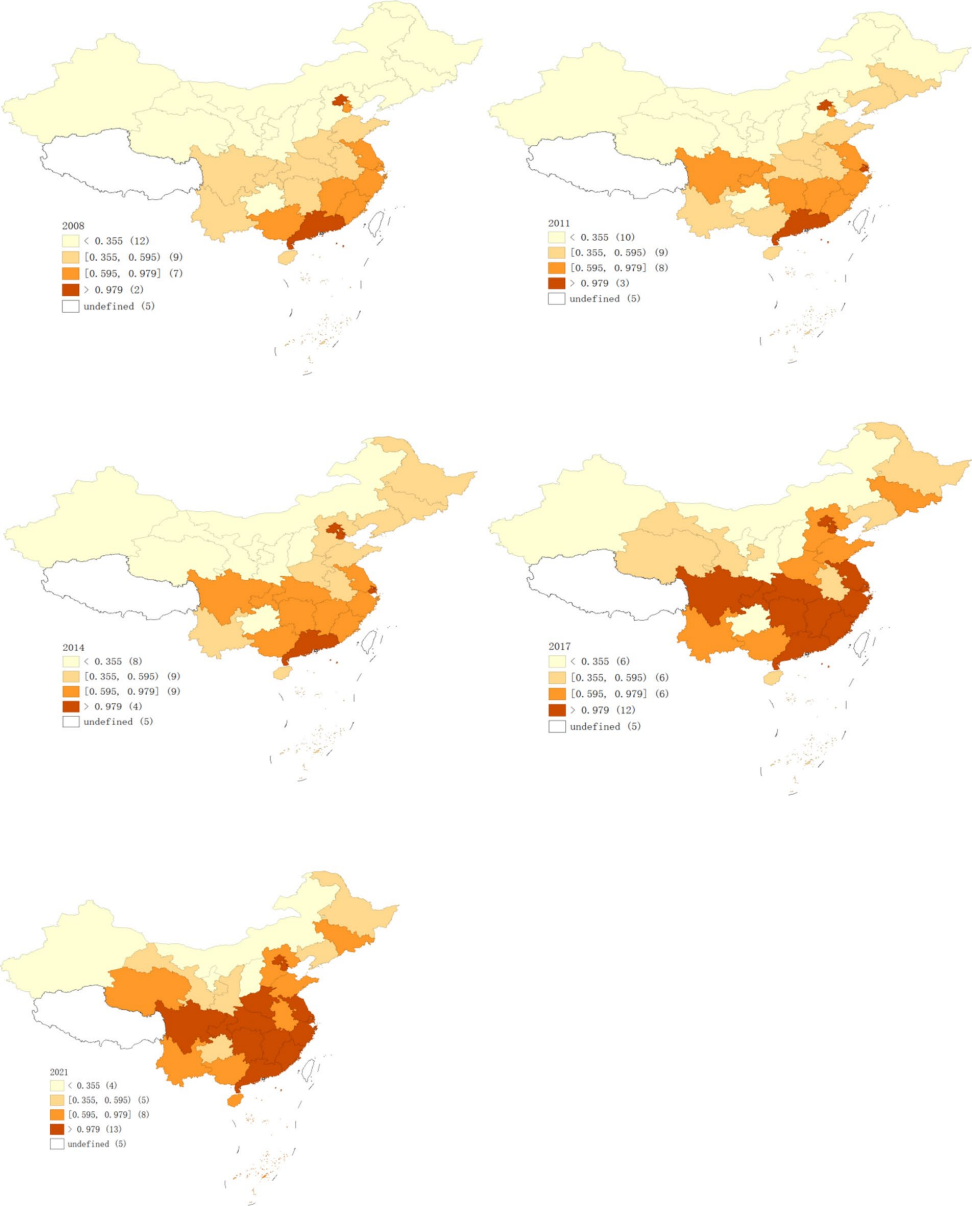
Spatial distribution pattern of CP in 2008, 2011, 2014, 2017 and 2021. *Notes*: ① *Source*: Authors’ own drawing, based on GeoDa 1.22.0.4 software. ② Source of the map of China: GeoAtlas (areas_v3). Map Review Number: GS Jing (2022) No. 1061. Obtained from https://datav.aliyun.com/portal/school/atlas/area_selector; ③ Undefined are Tibet, Hong Kong, Macau, Taiwan, and the Nansha Islands, which are not included in the study scope.

As shown in Fig. 3, CP in China exhibited an upward trend from 2008 to 2021. Beijing experienced the fastest growth, with CP increasing from 1205 CNY/ton in 2008 to 4105 CNY/ton in 2021, followed by Chongqing and Shanghai, where PC grew by 1303 CNY/ton and 1132 CNY/ton, respectively. These regions have successfully reduced carbon emissions while maintaining economic output. However, CP in Xinjiang and Shanxi has declined. The energy-dependent economic growth and lower energy efficiency in these regions have resulted in higher CO_2_ emissions per unit of GDP. The industrial structure in these provinces, which relies heavily on energy-intensive industries, presents a significant challenge to achieving carbon reduction targets.

The results presented in Table 3 indicate that the global Moran’s I for CP in each province of China is positive, signifying a significant spatial correlation. However, Moran’s I has demonstrated a decreasing trend over time, suggesting growing heterogeneity in CP across provinces also highlighted significant regional disparities in CP across China. The data reveal a pattern of “high in the east and low in the west, high in the south and low in the north, with rising levels in the center,” with CP growth being faster in the central region and slower in the western region.

**Table 3:** Global Moran’s I of CP for the years 2008–2021. *Source*: Authors’ owns estimation, based on Stata 14.0 software.

Year	I	Sd(I)	Z	P value
2008	0.3174	0.092	3.8267	0.0001
2009	0.3599	0.0916	4.3053	0.0000
2010	0.3274	0.0901	4.0153	0.0001
2011	0.3380	0.0883	4.2185	0.0000
2012	0.3511	0.0896	4.3031	0.0000
2013	0.3197	0.0879	4.0299	0.0001
2014	0.3538	0.0892	4.3518	0.0000
2015	0.3211	0.0884	4.0229	0.0001
2016	0.3373	0.0861	4.3192	0.0000
2017	0.3087	0.0841	4.082	0.0000
2018	0.2944	0.0847	3.8826	0.0001
2019	0.2669	0.084	3.5878	0.0003
2020	0.2399	0.0848	3.2369	0.0012
2021	0.2322	0.0811	3.2893	0.0010

To examine the spatial correlation of CP, this study utilizes global Moran’s I. The results presented in Table 3 indicate that the global Moran’s I for CP in each province of China is positive, signifying a significant spatial correlation. However, Moran’s I has demonstrated a decreasing trend over time, suggesting growing heterogeneity in CP across provinces.

### Spatiotemporal evolution patterns of high-quality economic development

**Fig. 4:**
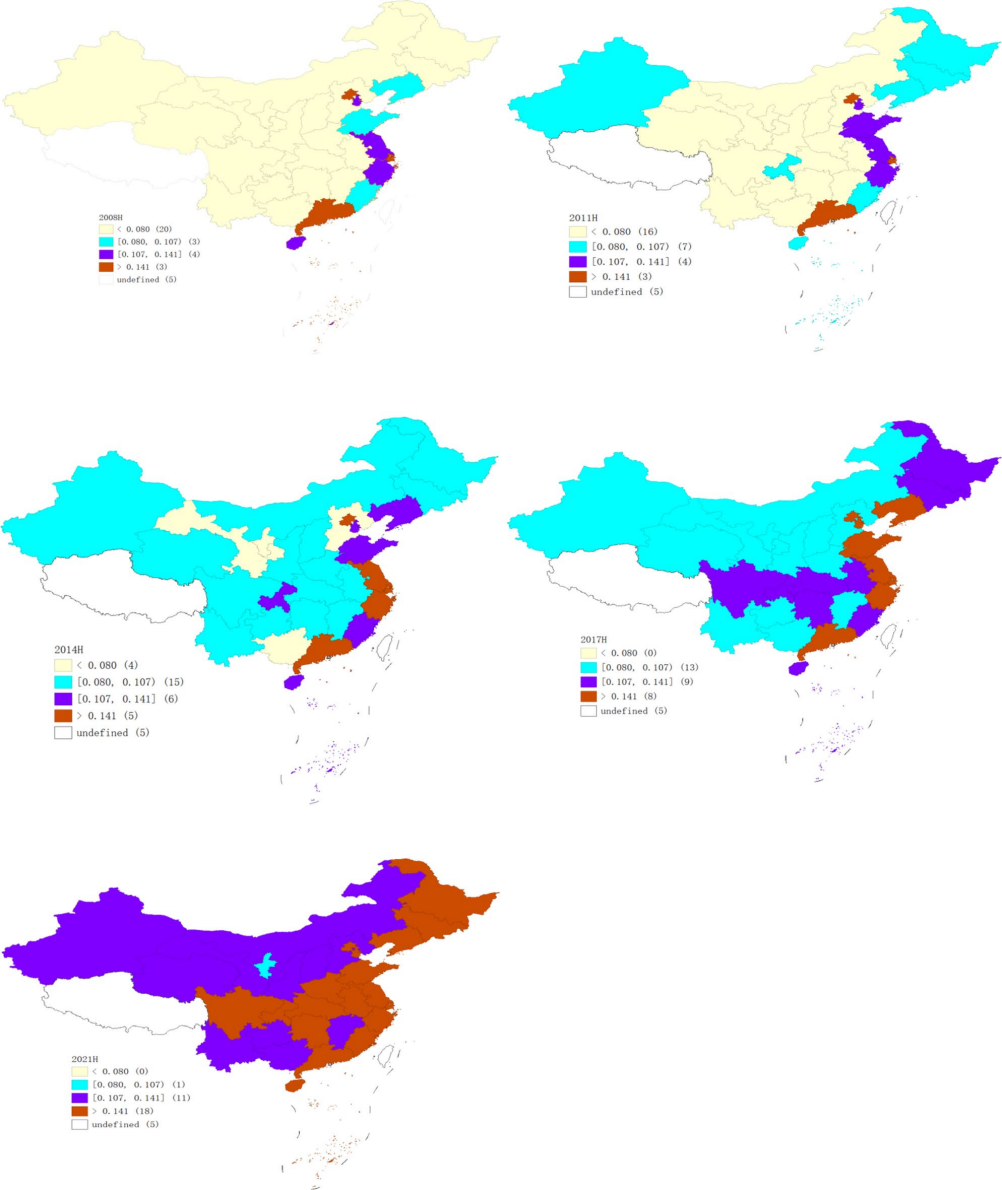
Spatial‒temporal evolution of HQED in 2008, 2011, 2014, 2017 and 2021. *Source*: Authors’ own calculations, using GeoDa 1.22.0.4 software.

The entropy method was employed to calculate the HQED index from 2008 and 2021, HQED consistently increased, with notable regional differences (see Fig. 4). The eastern region maintained a leading position, while the central and western regions exhibited more fluctuations. Some regions with initially high HQED declined, while others improved from a lower starting point. In 2008, provinces such as Hebei, Jiangxi, and Henan had lower HQED scores, and by 2021, provinces like Shanxi, Inner Mongolia, and Guizhou had some of the lowest scores.

### Spatiotemporal evolution patterns of coupling coordination degree

**Fig. 5:**
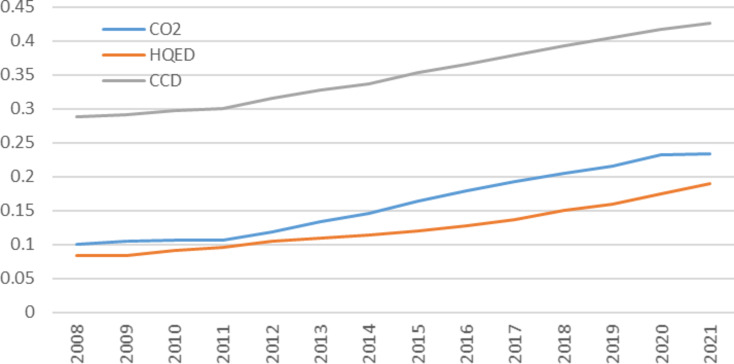
Trends of average values of CP, HQED, and CCD from 2008 to 2021**.** *Source*: Authors’ own calculations.

As shown in Fig. 5, from 2008 to 2021, CP, HQED, and CCD all increased. Before 2012, CP and HQED grew slowly. In 2011, severe natural disasters caused by climate change affected 430 million people and resulted in direct economic losses of 309.6 billion yuan. In response, the Chinese government made climate change a key focus in national planning and promoted green development. The 12th Five-Year Plan in 2012 aimed to advance environmental protection and foster a sustainable society. As a result, from 2013 onward, the growth rates of CP, HQED, and CCD significantly improved.

Table 4 presents the CCD from 2008to 2021, highlighting significant differences across provinces. Notably, all provinces, except Shanxi, shows a continuous upward trend. Provinces with higher levels of economic development tend to have higher CCD. As shown in Table 2, Beijing, Shanghai, and Guangdong have already reached the coordination stage.

**Table 4:** CCD of CP and HQED. *Source*: Authors’ own calculations, based on Excel and Stata software.

Province	2008	2011	2014	2017	2021
Beijing	0.5060	0.5507	0.6084	0.7076	0.8149
Shanghai	0.4645	0.4841	0.5329	0.5903	0.6475
Guangdong	0.4407	0.4511	0.4910	0.5427	0.6016
Jiangsu	0.3705	0.3836	0.4273	0.4810	0.5559
Tianjin	0.3691	0.3714	0.4236	0.4762	0.5250
Zhejiang	0.3580	0.3822	0.4256	0.4764	0.5160
Chongqing	0.2848	0.3204	0.3903	0.4490	0.5150
Hainan	0.3123	0.3052	0.3309	0.3557	0.5059
Hunan	0.2906	0.3122	0.3769	0.4167	0.5047
Sichuan	0.2866	0.3157	0.3727	0.4448	0.4938
Hubei	0.2986	0.3048	0.3743	0.4158	0.4667
Fujian	0.3579	0.3616	0.3931	0.4341	0.4539
Jiangxi	0.2848	0.3150	0.3576	0.3884	0.4382
Henan	0.2803	0.2626	0.3251	0.3565	0.4348
Shandong	0.2834	0.3144	0.3503	0.3854	0.4275
Jilin	0.2592	0.2767	0.3269	0.3582	0.4163
Guangxi	0.3088	0.2930	0.3359	0.3670	0.4081
Anhui	0.2541	0.2737	0.2962	0.3458	0.4067
Yunnan	0.2621	0.2861	0.3183	0.3570	0.4015
Hebei	0.2411	0.2528	0.2850	0.3381	0.3839
Liaoning	0.2702	0.2980	0.3315	0.3597	0.3701
Heilongjiang	0.2605	0.2623	0.3064	0.3366	0.3639
Qinghai	0.2247	0.2389	0.2638	0.3168	0.3549
Shaanxi	0.2154	0.2428	0.2576	0.2730	0.3236
Guizhou	0.2004	0.2150	0.2361	0.2602	0.3195
Gansu	0.2232	0.2359	0.2649	0.3007	0.3165
Inner Mongolia	0.2005	0.1986	0.2269	0.2591	0.2582
Xinjiang	0.2321	0.2197	0.2342	0.2430	0.2366
Ningxia	0.1401	0.1158	0.1608	0.1789	0.1856
Shanxi	0.1612	0.1712	0.0686	0.1397	0.1267

**Fig. 6:**
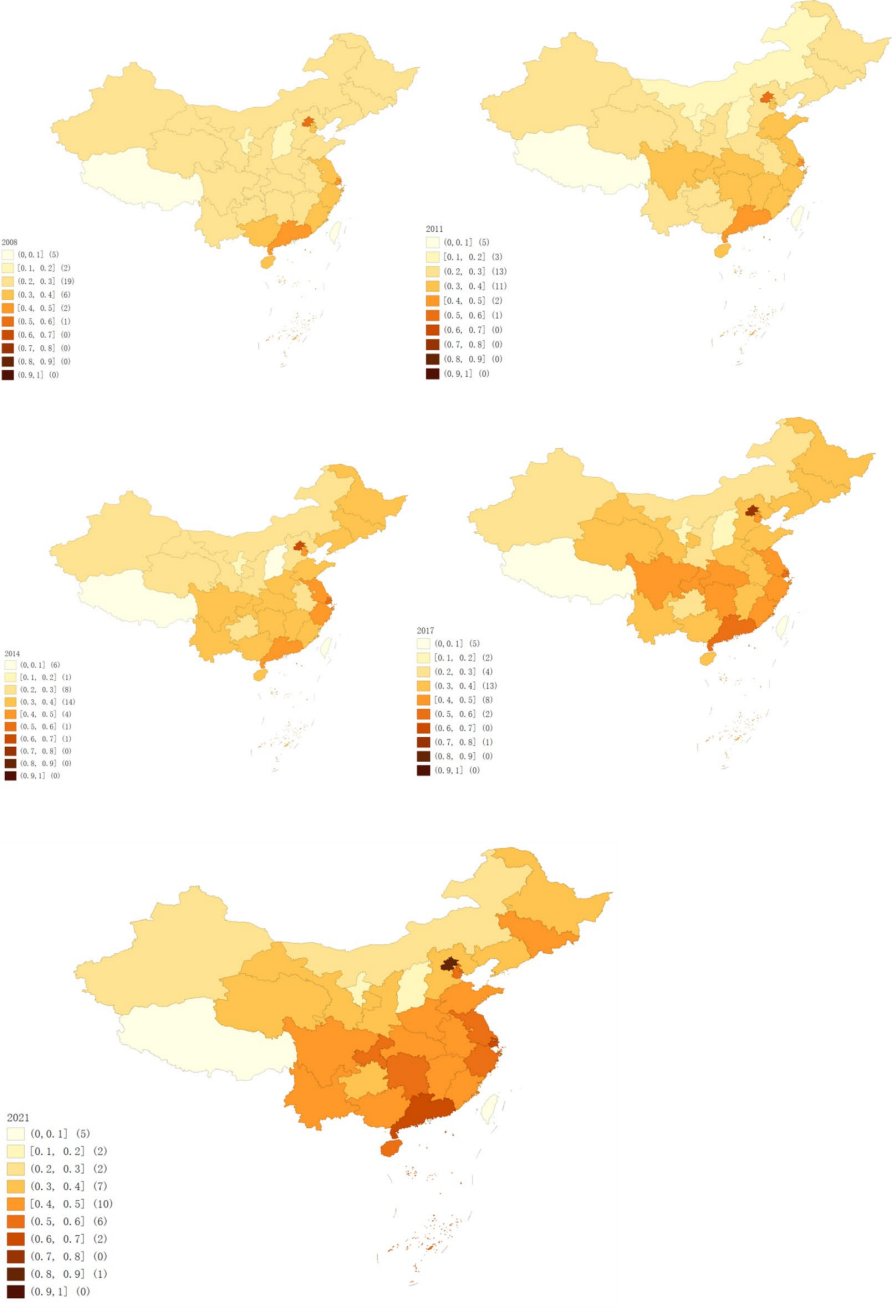
Spatial evolution of the CCD in 2008, 2011, 2014, 2017, and 2021. *Notes*: ① Source: Authors’ own drawing, based on GeoDa 1.22.0.4 software. ② The map of China includes Tibet, Hong Kong, Macau, Taiwan, and the Nansha Islands. These areas are not included in the study data, so the value of (0, 0.1] needs to be subtracted by 5.

Shanxi, known for its significant coal production, has leading industries in traditional heavy sectors such as coal, iron and steel, and chemicals, all of which are energy-intensive and high-emission^57^. In response to national policy shifts, the provincial government has implemented a series of environmental measures aimed at promoting green, low-carbon, and sustainable development. However, due to limited technological innovation and geographical constraints, Shanxi faces greater challenges in industrial upgrading and economic transformation compared to other provinces^58^. These factors have hindered province’s economic development, leading to a decline in its economic indicators. Figure 6 illustrates the CCD for 2008, 2011, 2014, 2017, and 2021. The quantile map, created using GeoDa, visually represents the spatial distribution and changes of the CCD over time, providing a clear view of its spatial and temporal evolution.

Figure 6 visualizes the regional changes in the CCD, revealing a pattern characterized by “high in the east and low in the west, high in the south and low in the north”. Between 2008 and 2011, 27 provinces exhibited a state of incoordination or coupling coordination, with some provinces, such as Jiangxi, Shandong, Hunan, and Hubei, experiencing a decline in the CCD. Beijing,

Shanghai, and Guangdong were in a state of adjustment. During this period, despite China’s high economic growth, the trend of coordinated development between HQED and CP was not particularly evident.

From 2011 to 2014, the number of provinces in a state of incoordination decreased from 27 to 24, while the number of provinces undergoing adjustments increased from 3 to 5. Only Beijing entered a stage of coordinated development during this period. In 2012, China’s GDP growth rate began to decline. In 2013, General Secretary Xi Jinping emphasized at the National Organizational Work Conference that judging success on the basis solely of GDP growth was no longer appropriate. In 2014, General Secretary Xi Jinping stated that China’s economy had entered a “new normal,” where the economic growth rate shifted from high to medium–high, with a focus on continuously optimizing the economic structure and prioritizing the quality of economic development.

Between 2014 and 2017, the CCD experienced significant changes, with the number of incoordination incidents decreasing from 24 to 19 and the number of provinces undergoing adjustments increasing from 5 to 10. During this period, the State Council issued action programs focused on energy conservation, emission reduction, and low-carbon development. With the continuous introduction of these policies, significant progress was made in both environmental protection and economic upgrading, leading to marked improvements in the coordination of these efforts.

Between 2017 and 2021, despite the global economic impact of the COVID-19 pandemic, the CCD in most provinces improved. The number of incoordination incidents decreased from 19 to 11, while the number of provinces in the adjustment phase increased from 10 to 16. Additionally, the number of provinces achieving coordination increased from 1 to 3. In 2017, the report from the19th CPC National Congress clearly stated that China’s economy had shifted from a high-speed growth stage to a high-quality development stage. During this period, China’s ecological environment significantly improved, regional development coordination and balance increased, and emissions per unit of CO_2_ steadily declined. In March 2020, China committed to achieving dual carbon goals as part of the Paris Agreement. This commitment was incorporated into the 14th Five-Year Plan and the 2035 Vision Outline^59^, providing clear guidance for China’s future direction in terms of carbon emissions and economic development.

### Data distribution characteristics

**Fig. 7:**
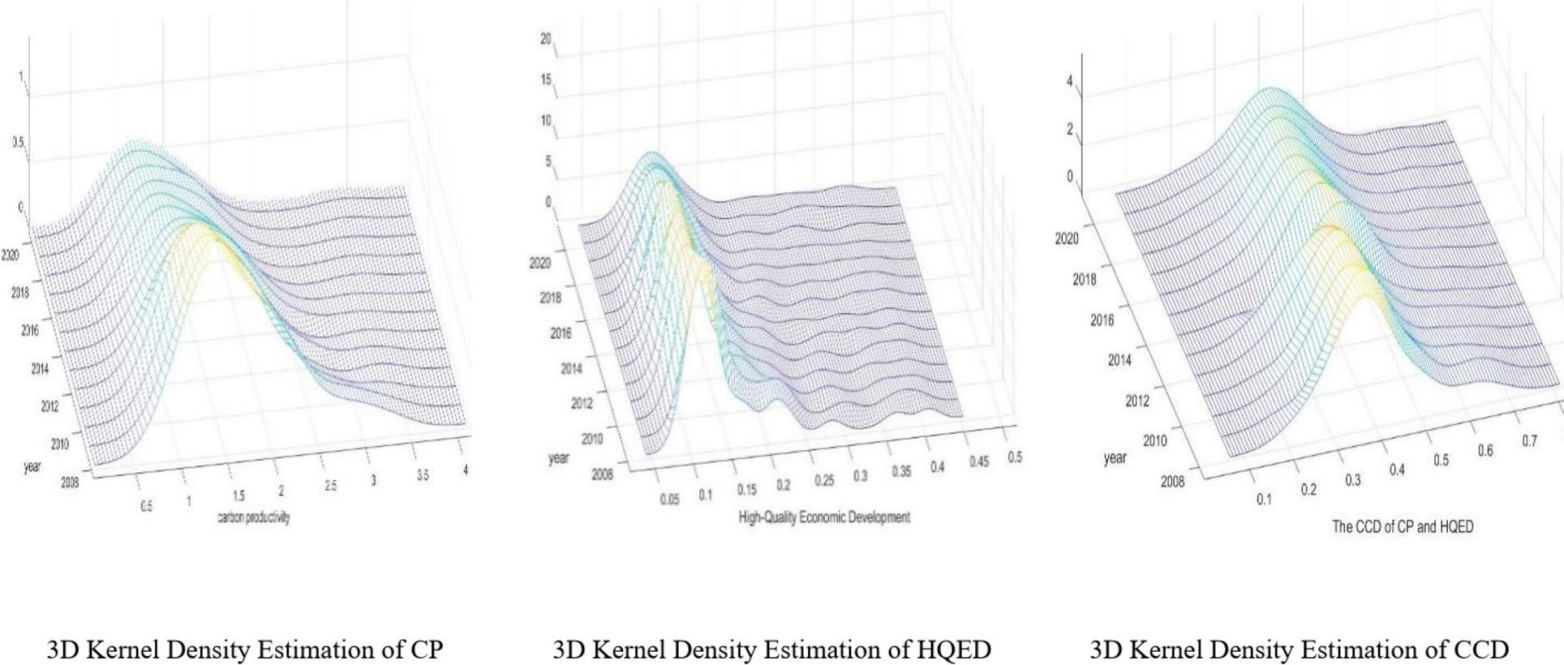
3D Kernel Density Estimation. *Source*: Authors’ own calculations, based on MATLAB software.

To more vividly and intuitively the levels, distribution evolution, extensibility, and polarization trends of CP, HQED, and the CCD between CP and HQED, the study employs 3D dynamic Kernal density estimation to generate Fig. 7.

As seen in Fig. 7, the kernel density curves of CP, HQED, and CCD exhibit a consistent pattern, all displaying an overall upward trend. In 2008, They all show sharp peaks, indicating that CP, HQED, and CCD were concentrated in the lower value regions at that time, reflecting weak synergy between economic development and carbon reduction. Following this, the sharp peaks gradually transition into wider peaks, suggesting that the polarization effect is weakening, meaning that CP, HQED, and CCD are improving synchronously, and the coupling development between the two is gradually strengthening. Additionally, the curves clearly exhibit a right-skewed tail, indicating that in some regions, CP, HQED, and CCD are significantly above average, with a few areas outperforming others. This suggests considerable regional disparity in the coupling coordination levels. The shape of the peaks in the kernel density map and their changes over time align with the analysis presented above.

### Spatial correlation analysis

Table 5 shows that from 2008 to 2021, the global Moran’s I values of the CCD are consistently greater than 0, with p-values consistently below 0.01, confirming statistical significance. This suggests that the CCD exhibits a strong positive spatial correlation and has significant global spatial clustering characteristics. Additionally, the trend in the Moran index over time reveals a gradual weakening of spatial correlation. This weakening indicates that GDP is progressively decoupling from CO_2_ emissions, thereby reducing the spatial correlation effect.

**Table 5:** Global Molan’s I of the CCD. *Source*: Authors’ own estimation, based on Stata software.

Year	I	Sd(I)	z	p value
2008	0.4119	0.0914	4.883	0.0000
2009	0.3995	0.0913	4.7514	0.0000
2010	0.3836	0.0911	4.5872	0.0000
2011	0.3828	0.0906	4.6058	0.0000
2012	0.3839	0.0912	4.5854	0.0000
2013	0.3749	0.0913	4.4817	0.0000
2014	0.3379	0.0907	4.1074	0.0000
2015	0.3360	0.0911	4.0681	0.0000
2016	0.3514	0.0911	4.2362	0.0000
2017	0.3311	0.0907	4.0294	0.0001
2018	0.3107	0.0907	3.8046	0.0001
2019	0.2803	0.0906	3.4734	0.0005
2020	0.2539	0.0910	3.1685	0.0015
2021	0.2393	0.0906	3.0202	0.0025

### Spatial agglomeration analysis

**Fig. 8:**
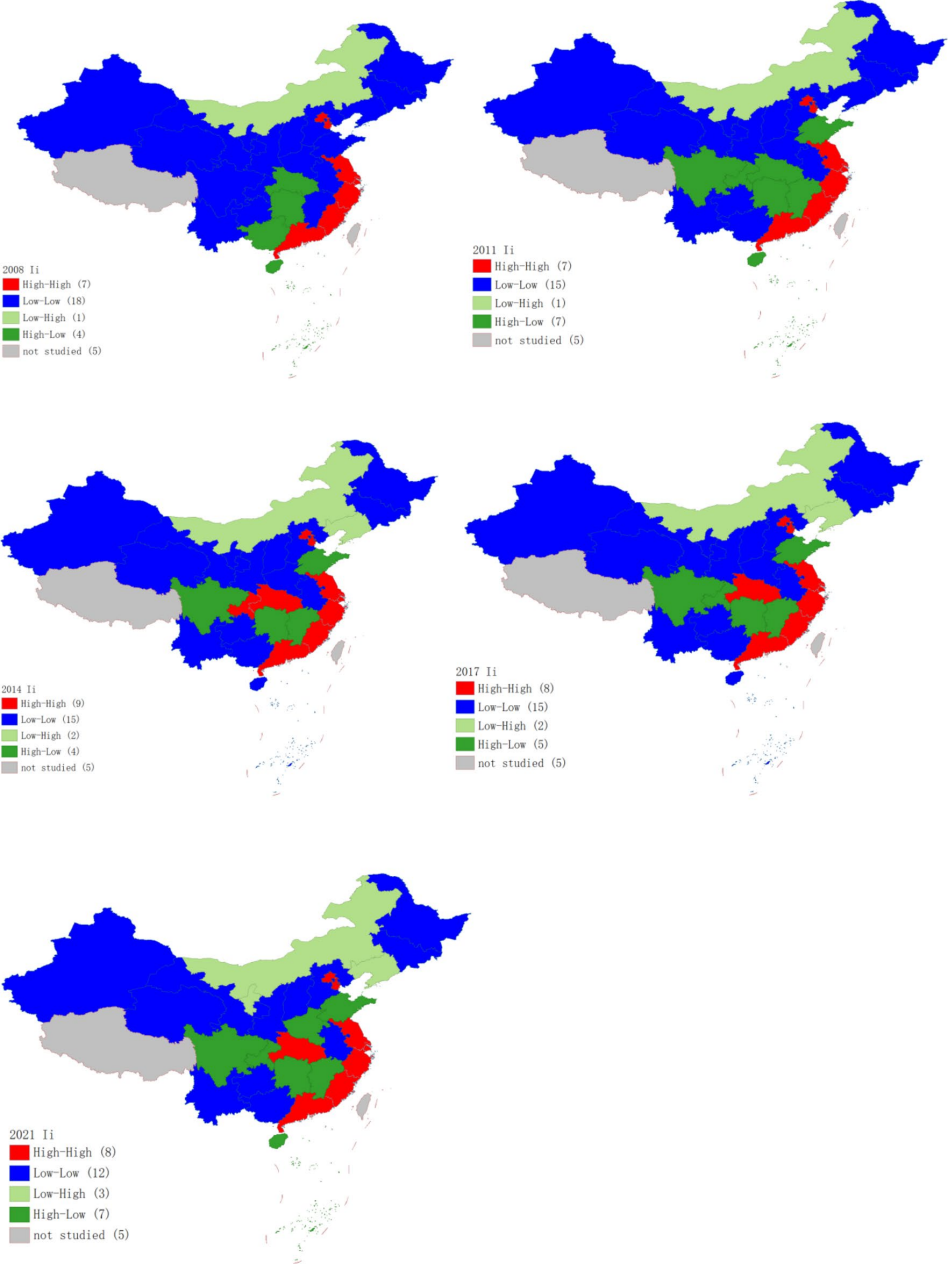
LISA chart of CCDs in 2008, 2011, 2014, 2017 and 2021. *Source*: Authors owns drawing, based on GeoDa 1.22.0.4 software.

To better understand the evolution of the CCD from 2008 to 2021, we concentrated on the years 2008, 2011, 2014, 2017, and 2021. Stata and GeoDa were employed to generate LISA agglomeration maps. Through the analysis of Fig. 8, we explored the spatial agglomeration patterns over time.

Between 2008 and 2021, the “H–H” agglomeration areas of CCD were primarily concentrated in economically developed regions along the eastern coast, including Beijing, Tianjin, Shanghai, Jiangsu, Zhejiang, and Guangdong. The number of “H–H” areas increased in 2014, 2017, and 2021.

The number of “L–L” agglomerations is higher in provinces with high energy consumption and relatively underdeveloped economies. These include Heilongjiang, Liaoning, and Jilin in Northeast China, as well as Shanxi, Hebei, Guizhou, Yunnan, Shaanxi, Gansu, Qinghai, Ningxia, and Xinjiang. These regions exhibit significant spatial incoherence between CP and HQED. The low-value clustering areas reflect limited economic influence on neighboring provinces, highlighting a lack of advantage in fostering HQED. This underscores the regional disparities and challenges in the coordinated development of CP and HQED.

Meanwhile, the number of “H–L” agglomeration areas has increased, indicating that some provincial regions have achieved more coordinated development within their jurisdictions but have limited influence on surrounding areas.

### Spatial effect analysis of coupling coordination degree

#### Analysis of spatial spillover effects

Based on the strong positive spatial autocorrelation of the CCD between CP and HQED, further analysis is conducted using a spatial econometric model to verify the spatial effects of the CCD. First, the appropriateness of selecting a spatial panel model for estimation should be confirmed. As shown in Table 6, the Variance Inflation Factor (VIF) value is 2.75, indicating no multicollinearity. The Hausman test statistic is 35.55, with a p-value of 0.0000, suggesting that fixed effects are preferred over random effects. Both the LM and Robust LM tests pass the significance tests, indicating that the SDM is more appropriate for estimation than the SLM or SEM, these results confirm that the SDM does not degrade into either the SEM or SLM. By comparing the R2 and Log-Likelihood values, the SDM model with both time and space fixed effects is the most scientifically reasonable approach for analyzing the spatial heterogeneity of factors influencing the CCD.

**Table 6:** Estimation results of spatial econometric model for CCD.

Variables	OLS	SDM
*ur*	0.1305*** (2.74)	0.1362 (1.556)
*er*	0.0004 (0.67)	− 0.0007 (− 1.218)
*rd*	0.1957** (2,04)	0.4694*** (5.034)
*pgdp*	0.0121*** (7.92)	0.0067*** (3.791)
*si*	− 0.1239 *** (− 3.38)	− 0.1339*** (− 4.029)
*ml*	0 .1426*** (7.92)	0.0919*** (5.083)
*_cons*	0.1914*** (6.84)	0.4021*** (6.609)
W*ur		− 0.1439 (− 1.016)
W*er		0.0064*** (3.434)
W*rd		− 0.7231*** (− 3.479)
W*pgdp		0.0233*** (6.471)
W*si		− 0.2538*** (− 3.148)
W*ml		− 0.0462 (− 1.294)
*ρ*		− 0.2061*** (− 2.577)
*R*^2^	0.49	0.56
*LM-spatiallag*		139.618***
*LM-spatialerror*		226.738***
*Rubust LM-spatiallag*		20.532***
*Rubust LM-spatialerror*		107.653***
*Vif*	2.75	
*Hausman test*	35.55 ***	20.74
*Both fixed*	Yes	yes

Statistics at the significant level are shown in parentheses, *** *p* < 0.01, ** *p* < 0.05, * *p* < 0.1

From the econometric estimation results in Table 6, comparing the OLS model without considering spatial factors with the SDM model reveals that in the ordinary panel regression model, all variables except for environmental regulation pass the significance test at the 5% level. However, in the SDM model, both urbanization level and environmental regulation do not pass the significance test. There are significant differences in the regression coefficients and their significance between the two models.

The SDM results shows that the coefficient estimates of the spatial lag terms for environmental regulation (W*er), technological progress (W*rd), per capita GDP (W*pgdp), and the share of the secondary industry (W*si) are all significant at the 1% level. However, urbanization and marketization level do not pass the significance test, indicating that technological progress (W*rd) and economic development level (W*pgdp) in neighboring regions have a significant positive spatial spillover effect on the local coupling coordination level. In contrast, environmental regulation (W*er) and the share of the secondary industry (W*si) in neighboring areas have a significant negative spatial effect on the local coupling coordination level. The spatial impact of urbanization and marketization level on the local coupling coordination level is insignificant.

There is a significant spatial spillover effect in the CCD between CP and HQED. The ρ value of the SDM model for coupling coordination is − 2.577, which is significant at the 1% level. The main reason for this is that the CCD is still in its early stages. Regions with high coupling coordination tend to have a siphon effect on surrounding areas, while the radiation and demonstration effects on neighboring low-value areas have not yet fully materialized, thus resulting in a spatial negative effect.

#### Analysis of spatial effects

Table 7 demonstrates that the variables have significantly different effects on the CCD.

**Table 7:** Spatial effect decomposition of coupling coordination between CP and HQED.

Variables	Direct effect	Indirect effect	Total effect
*ur*	0.1448 (1.539)	− 0.1552 (− 1.132)	− 0.0104 (− 0.150)
*er*	− 0.0009 (− 1.565)	0.0058*** (3.680)	0.0049*** (3.481)
*rd*	0.5023*** (5.397)	− 0.7034*** (− 3.924)	− 0.2012 (− 1.336)
*pgdp*	0.0059*** (3.321)	0.0189*** (5.203)	0.0248*** (8.712)
*si*	− 0.1266*** (− 3.877)	− 0.1946** (− 2.547)	− 0.3212*** (− 4.170)
*ml*	0.0938*** (5.264)	− 0.0554* (− 1.803)	0.0385 (1.247)

Statistics at the significant level are shown in parentheses, *** *p* < 0.01, ** *p* < 0.05, * *p* < 0.1

From the perspective of the total effect, both economic development and environmental regulation have a significantly positive impact on the CCD. The proportion of the secondary industry exerts negative total effect on the CCD, while the urbanization level, marketization level, and technological progress do not have a significant spatial total effect on the CCD.

From the perspective of direct effects, Economic development, R&D investment, and marketization all have significant positive impacts on the CCD. Economic development is the primary driver of the CCD, significantly influencing local coordination and exerting a notable spatial spillover effect on neighboring regions. R&D investment supports local economic growth and industrial upgrading through technological innovation, knowledge accumulation, and human capital enhancement, playing a central role in fostering low-carbon economic growth. Meanwhile, marketization typically improves resource allocation efficiency, drives technological innovation, optimizes industrial structure, and enhances the policy environment. The interaction of these factors promotes the positive coupling coordination between CP and HQED. The share of the secondary industry has a significantly negative effect, reflecting the degree of regional industrialization. As industrialization progresses, CO2 emissions rise, and the quality of economic development improves more slowly, leading to a decline in the CCD. This is particularly evident in regions such as the three northeastern provinces and Shanxi.

From the perspective of indirect effects, except for the insignificant impact of urbanization level, all other variables have a significant impact on the CCD. Among these, environmental regulation and economic development have a positive effect, while R&D investment, marketization level, and the share of the secondary industry have a negative effect. Economic development is tightly interconnected between regions; the economic growth of one area positively influences neighboring regions through various channels, promoting their growth and development. The direct effect of environmental regulation is negative but insignificant, possibly due to short-term cost burdens, unreasonable industry structures, and the lag in industrial transformation. However, its indirect effect is significantly positive, reflecting the positive role of mechanisms such as policy spillover effects, technological diffusion, and regional collaboration in enhancing the CCD of neighboring regions. The negative indirect effect of R&D investment suggests that, in regional interactions, factors such as competition, resource siphoning, and technology protection may lead to uneven development, thereby hindering the CCD. The negative significant effect of the secondary industry share is primarily due to the high energy consumption and emissions associated with the secondary industry, as well as the suppressive effect on the environmental quality and economic development of neighboring regions through industrial transfer. While improvements in marketization can optimize local resource allocation and stimulate enterprise vitality, they also lead to increased regional competition, resource siphoning effects, the lag of industrial restructuring, and policy differences with spatial spillover effects, which negatively impact the CCD in surrounding areas.

## Discussion

This study thoroughly explores the spatiotemporal evolution patterns of the CCD between CP and HQED in China, revealing the process of their coordinated development and regional disparities. Through multidimensional spatiotemporal analysis, including CCD, three-dimensional kernel density estimation, spatial autocorrelation analysis, and spatial econometric models, the study provides a more comprehensive perspective for understanding the coupling relationship between CP and HQED.

### Spatiotemporal evolution and development trends

Temporal Dimension: The coupling coordination degree between carbon productivity and high-quality economic development shows a fluctuating upward trend over time, reflecting progress in balancing economic growth and carbon emission reduction in China. The growth rate varies across different stages, with slower improvements in the early stages and accelerated growth in later stages driven by policy support, technological advancements, and increased environmental awareness.

Spatial Dimension: Significant spatial heterogeneity is observed in the distribution of carbon productivity, high-quality economic development, and their coupling coordination degree. Regions with high carbon productivity and high-quality economic development are often concentrated in specific areas, such as the Yangtze River Delta and the Pearl River Delta. The eastern region, characterized by the rapid development of high-tech industries and continuous optimization of industrial structures, achieves a relatively high coupling coordination degree. In contrast, the western region, with a single economic structure and a high proportion of resource-intensive industries, exhibits a lower coupling degree between carbon productivity and high-quality economic development. This finding is consistent with previous research, which also highlights the regional disparities in China’s economic development and carbon productivity, particularly the east–west divide.

### Spatial autocorrelation and regional clustering

Spatial autocorrelation analysis reveals significant spatial clustering effects in CP, HQED, and their coupling coordination. “H–H” clustering regions are primarily concentrated in the developed eastern areas, where technological spillovers and industrial linkages drive mutual promotion between CP and HQED. In contrast, “L-L” clustering regions are found in relatively underdeveloped areas, including energy-dependent heavy industrial provinces, the three northeastern provinces, and parts of the central and western regions. This phenomenon underscores the uneven development across regions. This result corroborates previous studies that emphasize the spatial autocorrelation and clustering effects in China’s regional economic and environmental development. However, this research goes further by using spatial econometric models to quantify the spatial spillover effects, which have often been overlooked in earlier studies.

### Analysis of key influencing factors

In terms of variable selection, key variables were chosen based on China’s unique economic development trajectory, with particular emphasis on domestic circulation, considering the unprecedented changes over the past century. Using a spatial econometric model, this study identifies urbanization level, environmental regulation, R&D investment, per capita GDP, and marketization level as critical factors influencing the CCD. The results indicate that economic development, environmental regulation, and the share of the secondary industry have the most significant total effects on the CCD, playing pivotal roles in driving coordinated development.

### Analysis of effect pathways

Compared to traditional ordinary least squares (OLS) methods, spatial econometric models provide a more comprehensive understanding of spatial relationships and interaction mechanisms between variables, reducing potential bias from ignoring spatial effects. Analyses of total effects, direct effects, and indirect effects reveal that variables not only exert direct impacts within their own regions but also influence neighboring regions through spatial spillover effects. This underscores the importance of considering the interactions and linkages between factors during policy implementation to maximize policy effectiveness.

### Limitations of the study

In comparison to earlier studies, which typically analyzed carbon productivity and economic development in isolation, this study combines both spatial econometrics and multidimensional variables to offer a more comprehensive approach. However, his study has certain limitations. In selecting indicators for high-quality economic development and the factors influencing the coupling coordination degree, although various aspects were considered, the potential issue of omitted variables remains. Future research could broaden the scope of influencing factors by incorporating variables from additional dimensions. Furthermore, while an economic-geographical weight matrix was used for the spatial weight matrix, future studies could refine the indicator system and explore more appropriate methods for constructing spatial weight matrices to improve research accuracy. Future studies could also extend the time span of the research and include forecasting analyses to explore the future relationship between the two. Additionally, investigating regional heterogeneity and examining the specific effects of policy interventions by region could provide more scientifically grounded insights for policy-making.

## Conclusion and policy implications

### Conclusion

1. *Significant regional development imbalance*. There are pronounced regional disparities in CP, HQED, and the CCD between the two, characterized by a spatial distribution pattern of “higher in the east and lower in the west.” This research conclusion aligns with previous literature and is closely linked to varying regional economic development levels, industrial structures, and resource endowments, thereby highlighting the imbalance in regional development across China.
2. *Strong spatial dependence*. Carbon productivity, high-quality economic development, and the coupling coordination degree between them exhibit strong spatial autocorrelation, indicating significant spillover effects between regions. This provides a theoretical basis for regional coordinated development and underscores the necessity of cross-regional cooperation and coordination.
3. *Multifactor influence on CCD*. Factors such as urbanization level, environmental regulation, R&D investment, per capita GDP, and marketization level collectively influence the coupling coordination degree. These factors exert both direct and indirect effects, impacting local areas and regions, thereby shaping the spatial pattern of the coupling coordination degree.
4. *Advantages of spatial econometric models*. Compared to traditional OLS regression, spatial econometric models excel in addressing spatial correlations between regions. They provide more accurate analyses of influencing factors and clearly reveal the profound impact of spatial spillover effects on the coupling coordination degree.

### Policy implications

1. *Formulate region-specific policies*. Regions should develop differentiated development strategies based on their resource endowments, industrial structures, and development stages. Eastern regions should continue advancing green technology innovation and high-tech industry development to consolidate carbon reduction achievements. Western regions should focus on strengthening green infrastructure construction, promoting industrial restructuring, and improving the coupling coordination degree.
2. *Strengthen regional collaboration*. Given the spatial spillover effects, cross-regional cooperation should be enhanced to facilitate the flow of technology, capital, and talent while establishing mechanisms for green development collaboration. In particular, complementary regional strengths in green industries, technologies, and the low-carbon economy should be fully leveraged. Initiatives such as jointly building industrial parks and fostering technological cooperation can enable resource sharing and synergy, promoting balanced improvements in carbon productivity and high-quality economic development across regions.
3. *Increase R&D investment and deepen market reforms*. Governments should enhance support for technological innovation and incentivize enterprises to increase R&D investment in green technologies and low-carbon economy sectors. Simultaneously, market reforms should be deepened to improve market efficiency and optimize resource allocation. Joint innovation among industries, academia, and research institutions should be promoted, alongside establishing a more robust intellectual property protection system to stimulate corporate innovation and accelerate the development of green technologies.
4. *Optimize environmental regulation policies*. While strengthening environmental regulations to enhance carbon productivity, it is essential to consider regional economic capacity and the practical challenges of industrial transformation. Flexible environmental policies should be implemented to balance carbon reduction and economic growth objectives. In particular, the overdevelopment of high-energy-consumption and high-pollution industries should be avoided in central and western regions.
5. *Improve monitoring and evaluation systems*. Develop a comprehensive evaluation system for carbon productivity and high-quality economic development, ensuring regular monitoring of regional indicator changes. Timely policy adjustments should be made based on these evaluations to enhance the precision and effectiveness of policy implementation.

## Data Availability

The data used in this study will be available upon request. Please contact the author via email at: 21010362@siswa.unimas.my.
